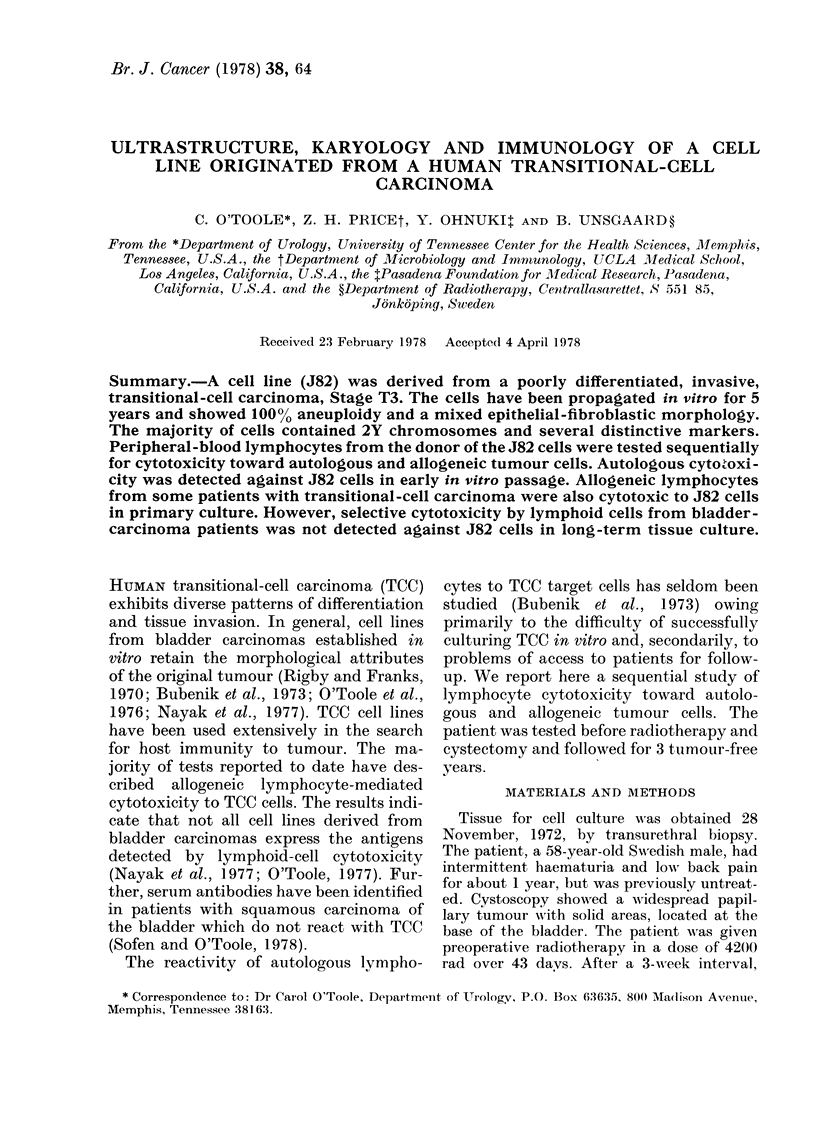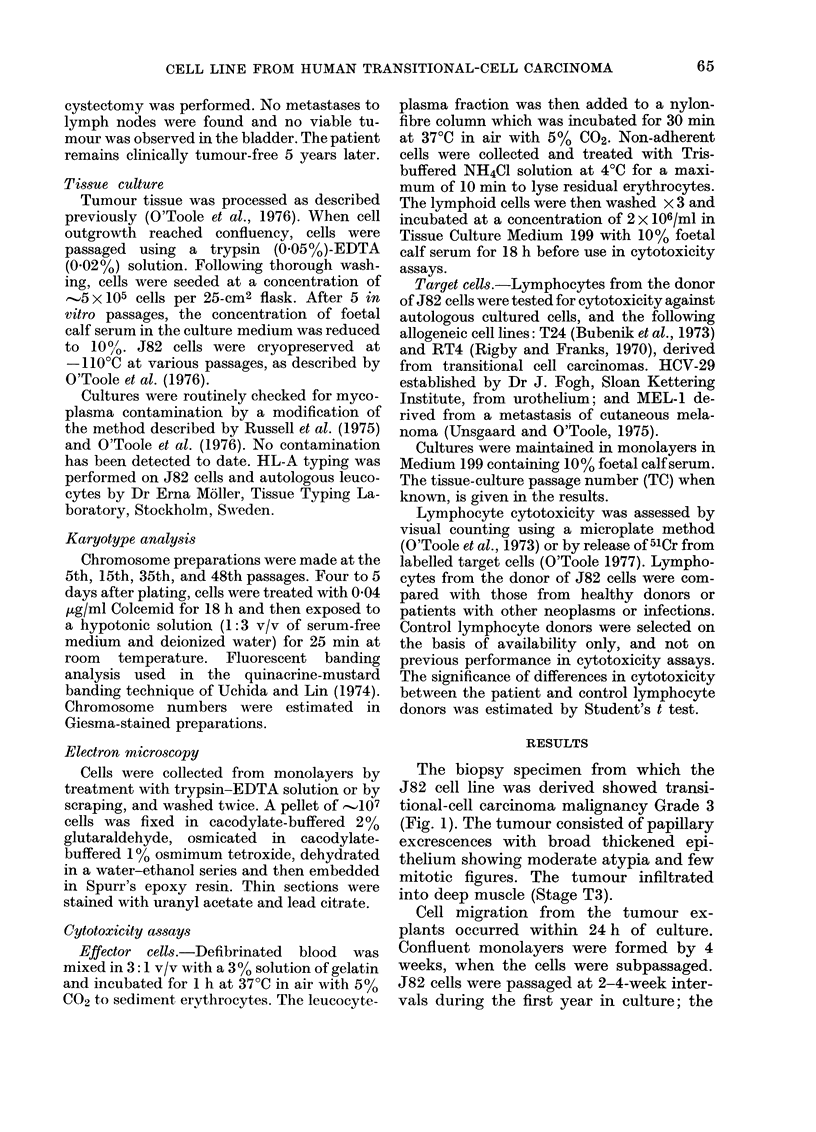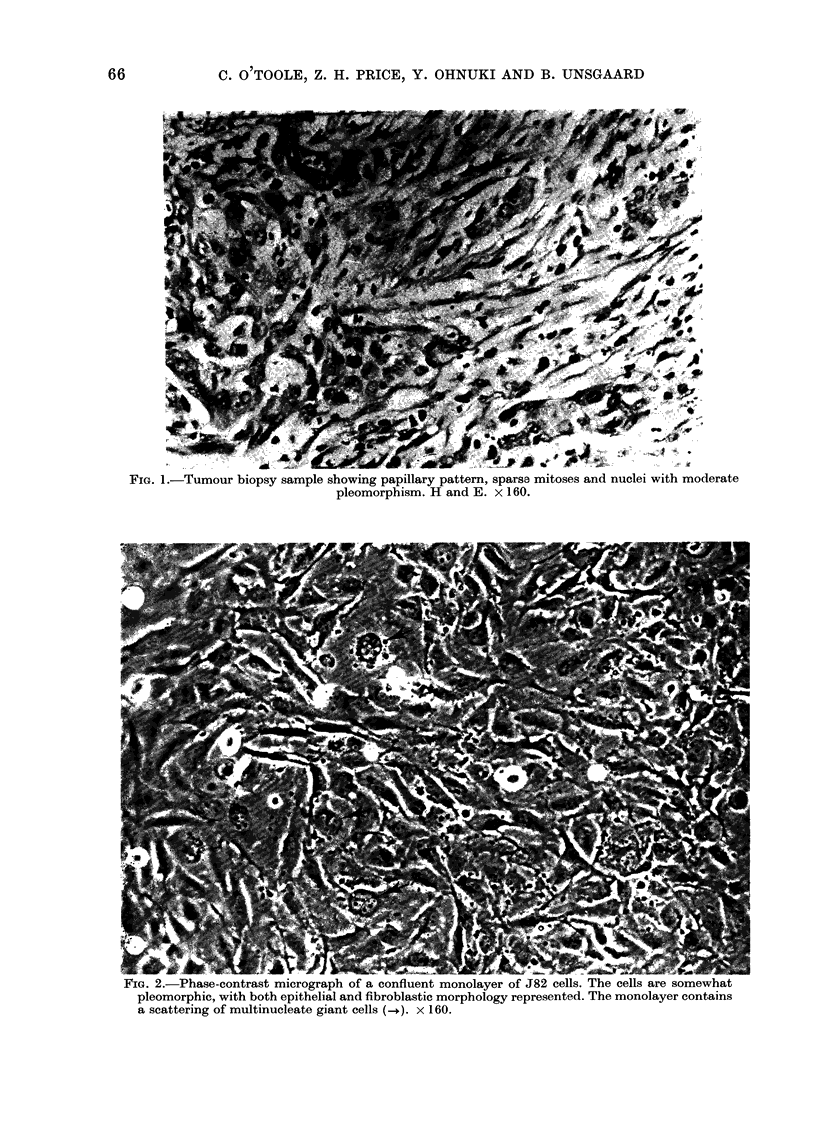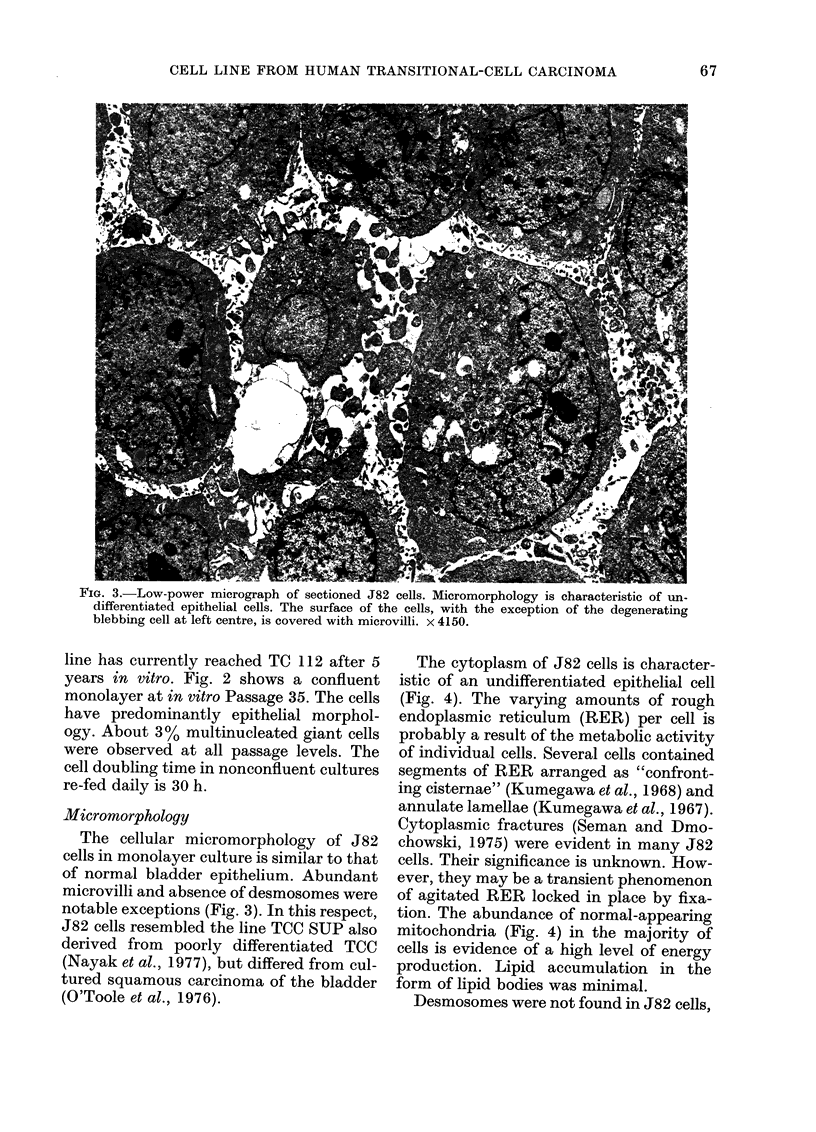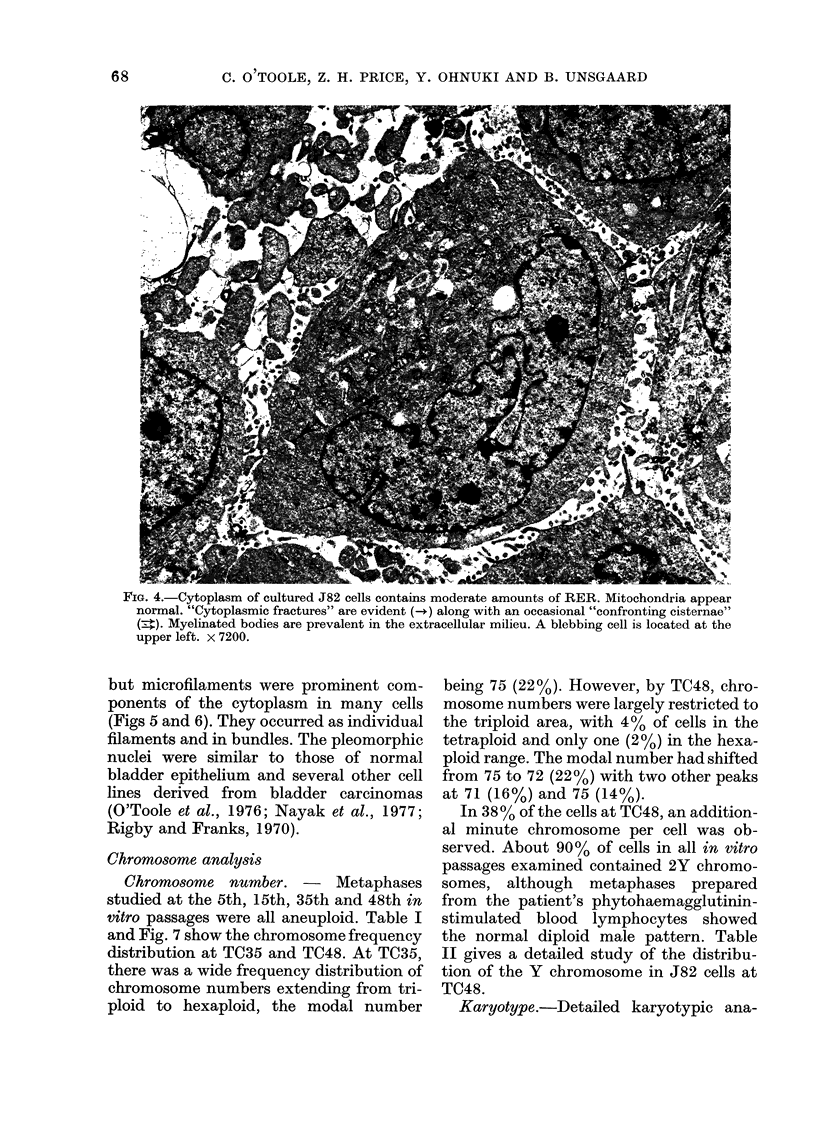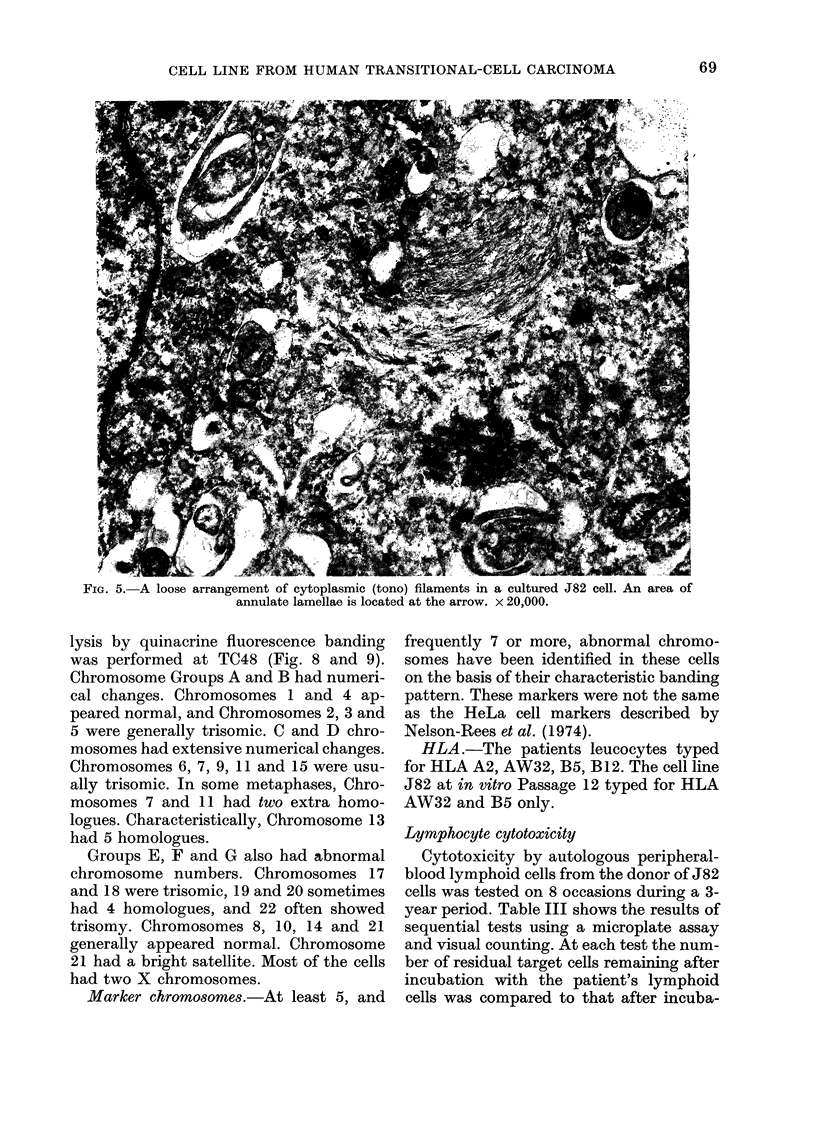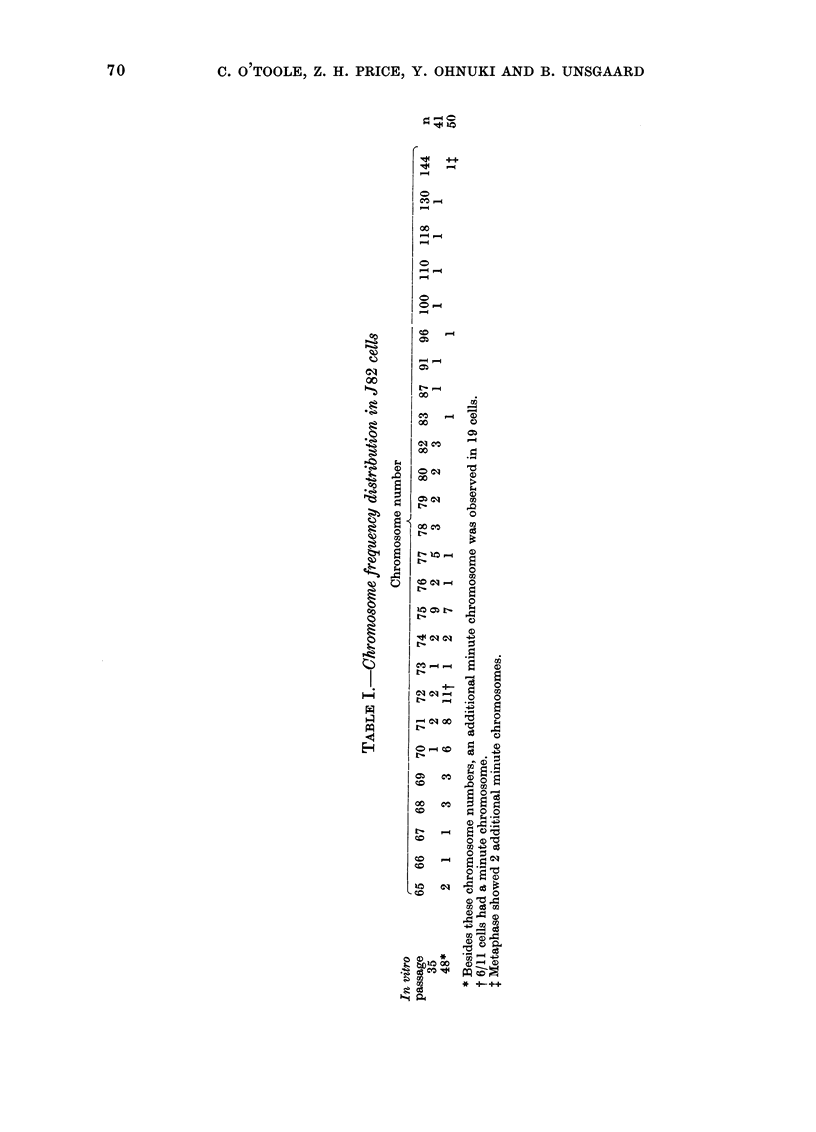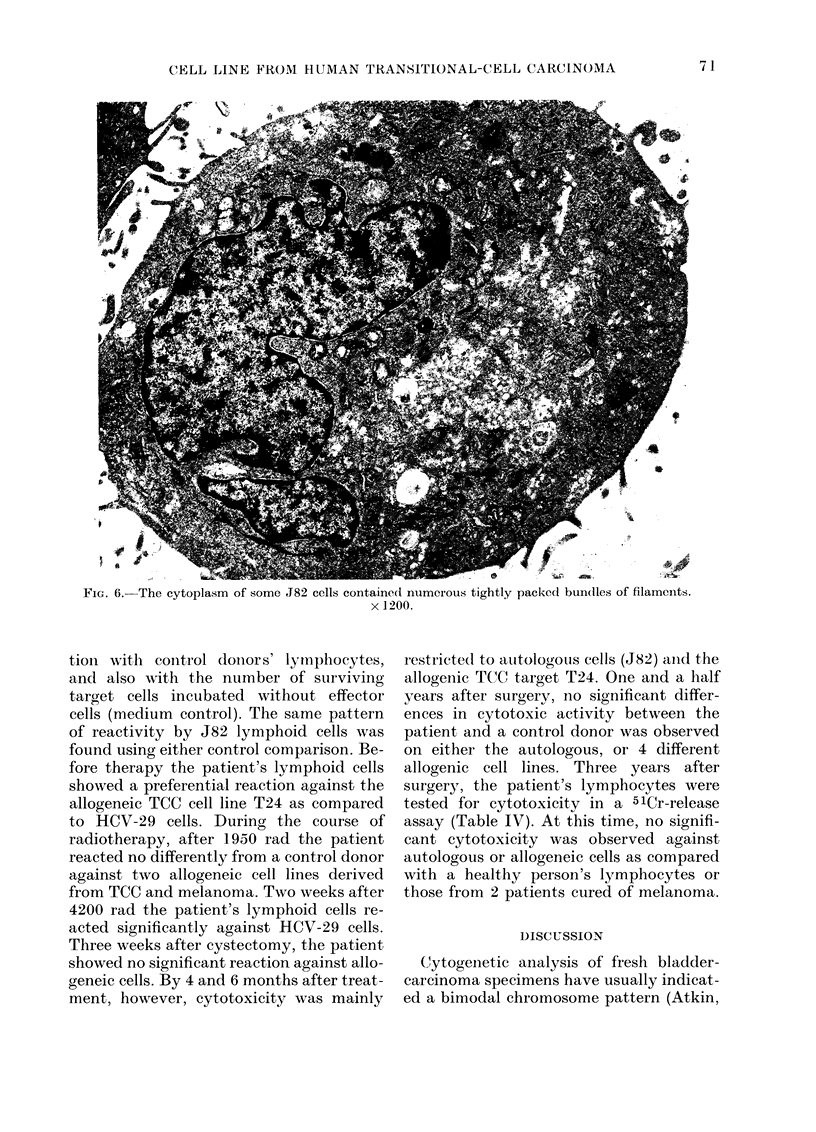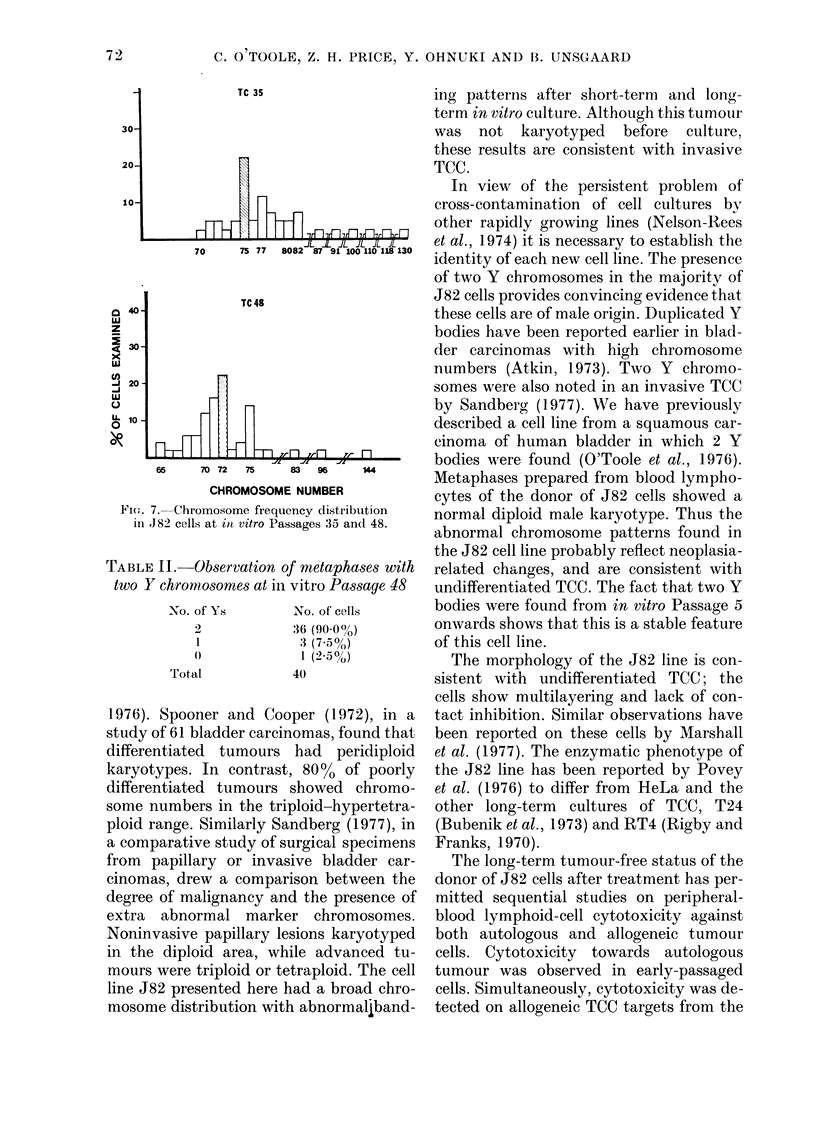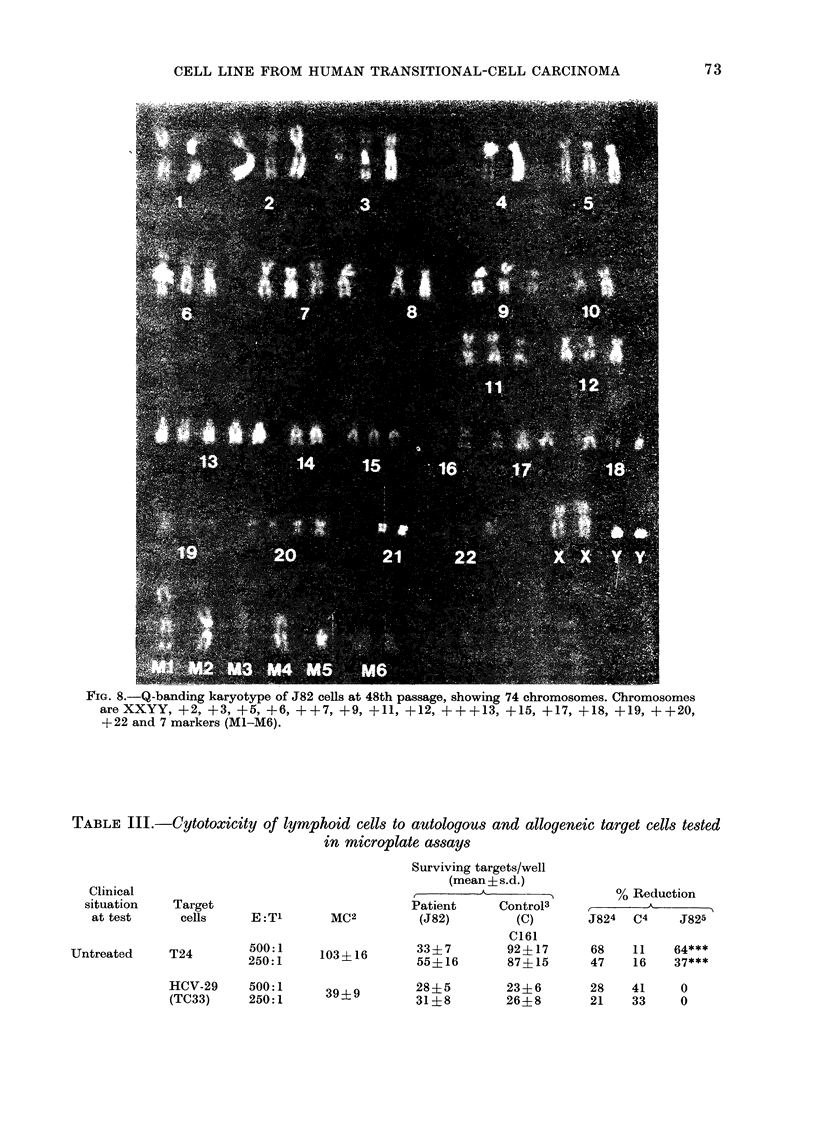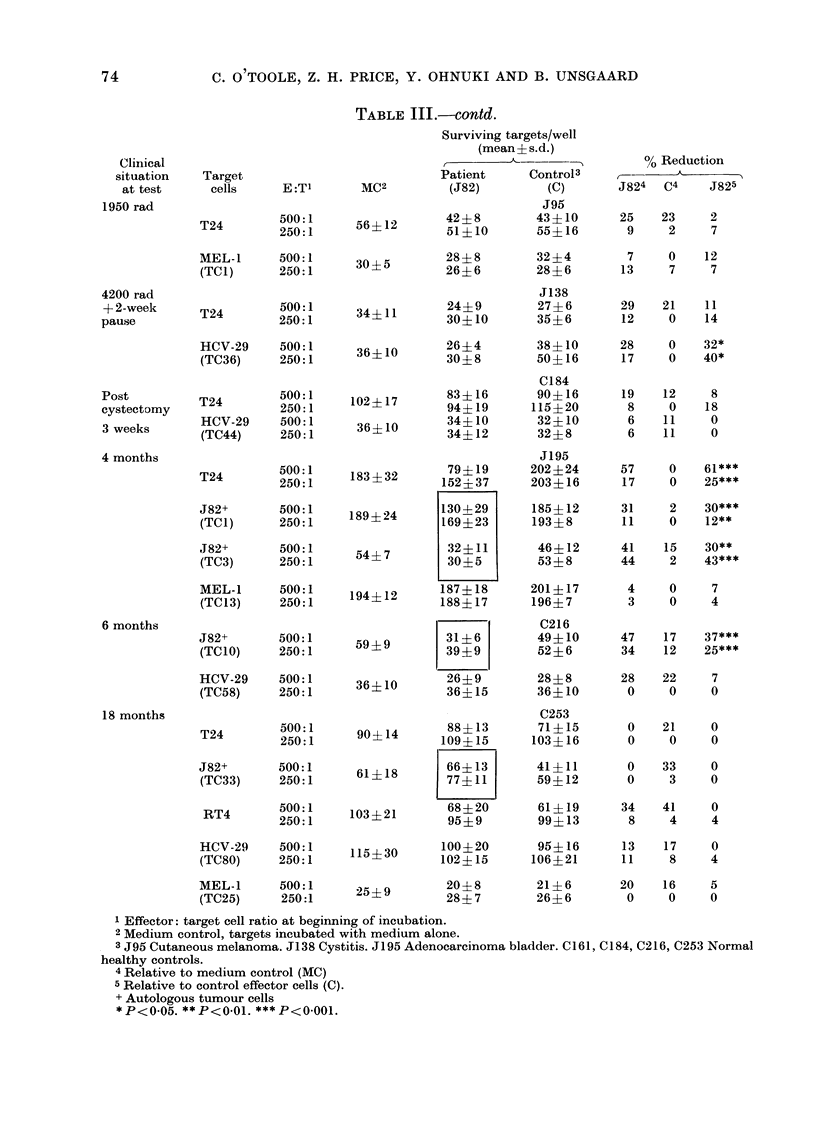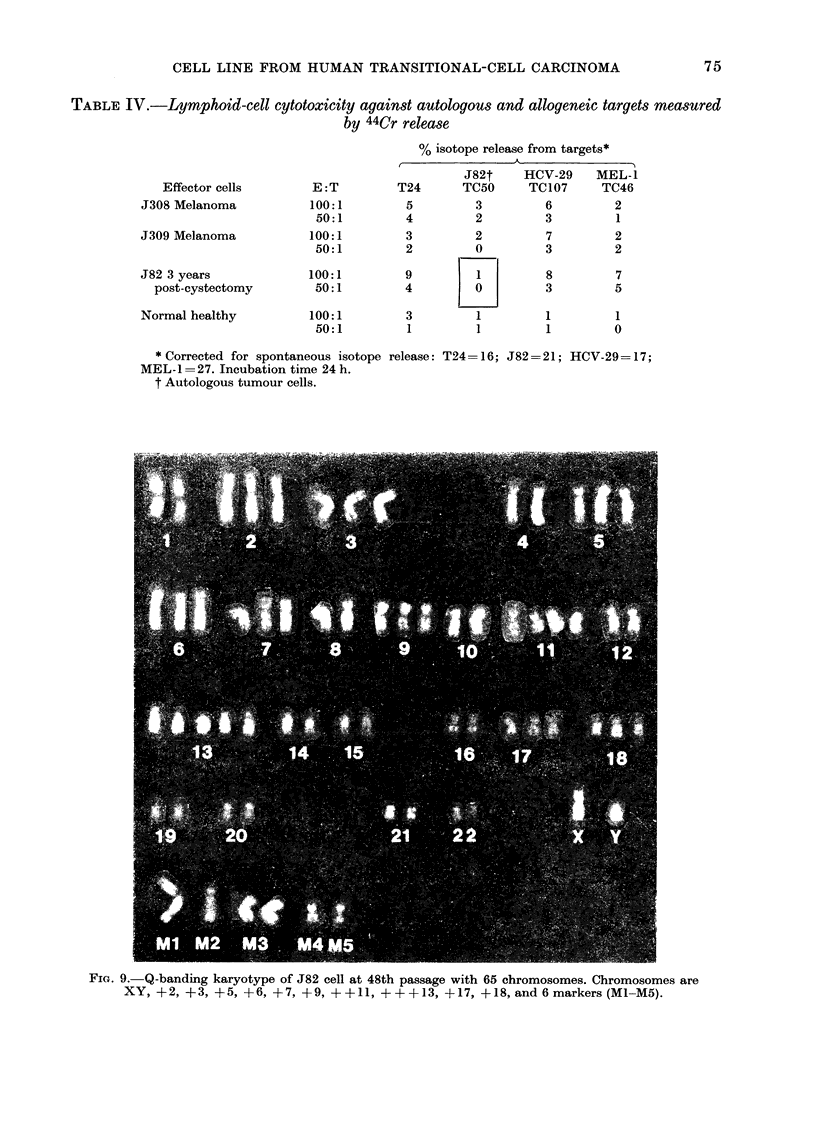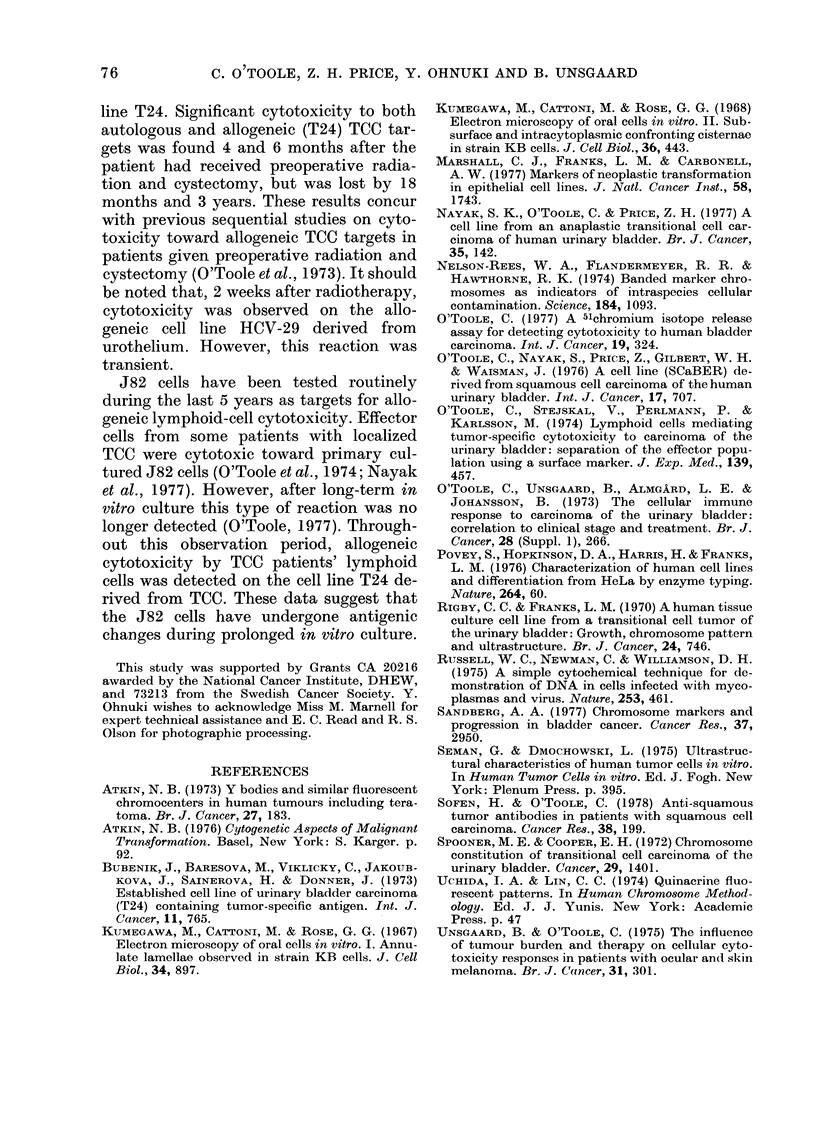# Ultrastructure, karyology and immunology of a cell line originated from a human transitional-cell carcinoma.

**DOI:** 10.1038/bjc.1978.164

**Published:** 1978-07

**Authors:** C. O'Toole, Z. H. Price, Y. Ohnuki, B. Unsgaard

## Abstract

**Images:**


*Br. J. Cancer* (1978) **38**, 64

### ULTRASTRUCTURE, KARYOLOGY AND IMMUNOLOGY OF A CELL LINE ORIGINATED FROM A HUMAN TRANSITIONAL-CELL CARCINOMA

C. O'TOOLE*, Z. H. PRICE†, Y. OHNUKI‡ AND B. UNSGAARD§

*From the *Department of Urology, University of Tennessee Center for the Health Sciences, Memphis, Tennessee, U.S.A., the †Department of Microbiology and Immunology, UCLA Medical School, Los Angeles, California, U.S.A., the ‡Pasadena Foundation for Medical Research, Pasadena, California, U.S.A. and the §Department of Radiotherapy, Centrallasarettet, S 551 85, Jönköping, Sweden*

Received 23 February 1978 Accepted 4 April 1978

**Summary.—A cell line (J82) was derived from a poorly differentiated, invasive, transitional-cell carcinoma, Stage T3. The cells have been propagated *in vitro* for 5 years and showed 100% aneuploidy and a mixed epithelial-fibroblastic morphology. The majority of cells contained 2Y chromosomes and several distinctive markers. Peripheral-blood lymphocytes from the donor of the J82 cells were tested sequentially for cytotoxicity toward autologous and allogeneic tumour cells. Autologous cytotoxicity was detected against J82 cells in early *in vitro* passage. Allogeneic lymphocytes from some patients with transitional-cell carcinoma were also cytotoxic to J82 cells in primary culture. However, selective cytotoxicity by lymphoid cells from bladder-carcinoma patients was not detected against J82 cells in long-term tissue culture.**

HUMAN transitional-cell carcinoma (TCC) exhibits diverse patterns of differentiation and tissue invasion. In general, cell lines from bladder carcinomas established *in vitro* retain the morphological attributes of the original tumour (Rigby and Franks, 1970; Bubenik *et al.*, 1973; O'Toole *et al.*, 1976; Nayak *et al.*, 1977). TCC cell lines have been used extensively in the search for host immunity to tumour. The majority of tests reported to date have described allogeneic lymphocyte-mediated cytotoxicity to TCC cells. The results indicate that not all cell lines derived from bladder carcinomas express the antigens detected by lymphoid-cell cytotoxicity (Nayak *et al.*, 1977; O'Toole, 1977). Further, serum antibodies have been identified in patients with squamous carcinoma of the bladder which do not react with TCC (Sofen and O'Toole, 1978).

The reactivity of autologous lymphocytes to TCC target cells has seldom been studied (Bubenik *et al.*, 1973) owing primarily to the difficulty of successfully culturing TCC *in vitro* and, secondarily, to problems of access to patients for follow-up. We report here a sequential study of lymphocyte cytotoxicity toward autologous and allogeneic tumour cells. The patient was tested before radiotherapy and cystectomy and followed for 3 tumour-free years.

### MATERIALS AND METHODS

Tissue for cell culture was obtained 28 November, 1972, by transurethral biopsy. The patient, a 58-year-old Swedish male, had intermittent haematuria and low back pain for about 1 year, but was previously untreated. Cystoscopy showed a widespread papillary tumour with solid areas, located at the base of the bladder. The patient was given preoperative radiotherapy in a dose of 4200 rad over 43 days. After a 3-week interval,

* Correspondence to: Dr Carol O'Toole, Department of Urology, P.O. Box 63635, 800 Madison Avenue, Memphis, Tennessee 38163.

cystectomy was performed. No metastases to lymph nodes were found and no viable tumour was observed in the bladder. The patient remains clinically tumour-free 5 years later.

*Tissue culture*

Tumour tissue was processed as described previously (O'Toole *et al.*, 1976). When cell outgrowth reached confluency, cells were passaged using a trypsin (0·05%)-EDTA (0·02%) solution. Following thorough washing, cells were seeded at a concentration of $\sim 5\times10^5$ cells per 25-$cm^2$ flask. After 5 *in vitro* passages, the concentration of foetal calf serum in the culture medium was reduced to 10%. J82 cells were cryopreserved at −110°C at various passages, as described by O'Toole *et al.* (1976).

Cultures were routinely checked for mycoplasma contamination by a modification of the method described by Russell *et al.* (1975) and O'Toole *et al.* (1976). No contamination has been detected to date. HL-A typing was performed on J82 cells and autologous leucocytes by Dr Erna Möller, Tissue Typing Laboratory, Stockholm, Sweden.

*Karyotype analysis*

Chromosome preparations were made at the 5th, 15th, 35th, and 48th passages. Four to 5 days after plating, cells were treated with 0·04 μg/ml Colcemid for 18 h and then exposed to a hypotonic solution (1:3 v/v of serum-free medium and deionized water) for 25 min at room temperature. Fluorescent banding analysis used in the quinacrine-mustard banding technique of Uchida and Lin (1974). Chromosome numbers were estimated in Giesma-stained preparations.

*Electron microscopy*

Cells were collected from monolayers by treatment with trypsin–EDTA solution or by scraping, and washed twice. A pellet of $\sim 10^7$ cells was fixed in cacodylate-buffered 2% glutaraldehyde, osmicated in cacodylate-buffered 1% osmimum tetroxide, dehydrated in a water–ethanol series and then embedded in Spurr's epoxy resin. Thin sections were stained with uranyl acetate and lead citrate.

*Cytotoxicity assays*

*Effector cells.*—Defibrinated blood was mixed in 3:1 v/v with a 3% solution of gelatin and incubated for 1 h at 37°C in air with 5% $CO_2$ to sediment erythrocytes. The leucocyte-plasma fraction was then added to a nylon-fibre column which was incubated for 30 min at 37°C in air with 5% $CO_2$. Non-adherent cells were collected and treated with Tris-buffered $NH_4Cl$ solution at 4°C for a maximum of 10 min to lyse residual erythrocytes. The lymphoid cells were then washed ×3 and incubated at a concentration of $2\times10^6$/ml in Tissue Culture Medium 199 with 10% foetal calf serum for 18 h before use in cytotoxicity assays.

*Target cells.*—Lymphocytes from the donor of J82 cells were tested for cytotoxicity against autologous cultured cells, and the following allogeneic cell lines: T24 (Bubenik *et al.*, 1973) and RT4 (Rigby and Franks, 1970), derived from transitional cell carcinomas. HCV-29 established by Dr J. Fogh, Sloan Kettering Institute, from urothelium; and MEL-1 derived from a metastasis of cutaneous melanoma (Unsgaard and O'Toole, 1975).

Cultures were maintained in monolayers in Medium 199 containing 10% foetal calf serum. The tissue-culture passage number (TC) when known, is given in the results.

Lymphocyte cytotoxicity was assessed by visual counting using a microplate method (O'Toole *et al.*, 1973) or by release of $^{51}Cr$ from labelled target cells (O'Toole 1977). Lymphocytes from the donor of J82 cells were compared with those from healthy donors or patients with other neoplasms or infections. Control lymphocyte donors were selected on the basis of availability only, and not on previous performance in cytotoxicity assays. The significance of differences in cytotoxicity between the patient and control lymphocyte donors was estimated by Student's *t* test.

### RESULTS

The biopsy specimen from which the J82 cell line was derived showed transitional-cell carcinoma malignancy Grade 3 (Fig. 1). The tumour consisted of papillary excrescences with broad thickened epithelium showing moderate atypia and few mitotic figures. The tumour infiltrated into deep muscle (Stage T3).

Cell migration from the tumour explants occurred within 24 h of culture. Confluent monolayers were formed by 4 weeks, when the cells were subpassaged. J82 cells were passaged at 2–4-week intervals during the first year in culture; the

FIG. 1.—Tumour biopsy sample showing papillary pattern, sparse mitoses and nuclei with moderate pleomorphism. H and E. ×160.

FIG. 2.—Phase-contrast micrograph of a confluent monolayer of J82 cells. The cells are somewhat pleomorphic, with both epithelial and fibroblastic morphology represented. The monolayer contains a scattering of multinucleate giant cells (→). ×160.

FIG. 3.—Low-power micrograph of sectioned J82 cells. Micromorphology is characteristic of undifferentiated epithelial cells. The surface of the cells, with the exception of the degenerating blebbing cell at left centre, is covered with microvilli. ×4150.

line has currently reached TC 112 after 5 years *in vitro*. Fig. 2 shows a confluent monolayer at *in vitro* Passage 35. The cells have predominantly epithelial morphology. About 3% multinucleated giant cells were observed at all passage levels. The cell doubling time in nonconfluent cultures re-fed daily is 30 h.

*Micromorphology*

The cellular micromorphology of J82 cells in monolayer culture is similar to that of normal bladder epithelium. Abundant microvilli and absence of desmosomes were notable exceptions (Fig. 3). In this respect, J82 cells resembled the line TCC SUP also derived from poorly differentiated TCC (Nayak *et al.*, 1977), but differed from cultured squamous carcinoma of the bladder (O'Toole *et al.*, 1976).

The cytoplasm of J82 cells is characteristic of an undifferentiated epithelial cell (Fig. 4). The varying amounts of rough endoplasmic reticulum (RER) per cell is probably a result of the metabolic activity of individual cells. Several cells contained segments of RER arranged as "confronting cisternae" (Kumegawa *et al.*, 1968) and annulate lamellae (Kumegawa *et al.*, 1967). Cytoplasmic fractures (Seman and Dmochowski, 1975) were evident in many J82 cells. Their significance is unknown. However, they may be a transient phenomenon of agitated RER locked in place by fixation. The abundance of normal-appearing mitochondria (Fig. 4) in the majority of cells is evidence of a high level of energy production. Lipid accumulation in the form of lipid bodies was minimal.

Desmosomes were not found in J82 cells,

FIG. 4.—Cytoplasm of cultured J82 cells contains moderate amounts of RER. Mitochondria appear normal. "Cytoplasmic fractures" are evident (→) along with an occasional "confronting cisternae" (⇉). Myelinated bodies are prevalent in the extracellular milieu. A blebbing cell is located at the upper left. × 7200.

but microfilaments were prominent components of the cytoplasm in many cells (Figs 5 and 6). They occurred as individual filaments and in bundles. The pleomorphic nuclei were similar to those of normal bladder epithelium and several other cell lines derived from bladder carcinomas (O'Toole *et al.*, 1976; Nayak *et al.*, 1977; Rigby and Franks, 1970).

*Chromosome analysis*

*Chromosome number.* — Metaphases studied at the 5th, 15th, 35th and 48th *in vitro* passages were all aneuploid. Table I and Fig. 7 show the chromosome frequency distribution at TC35 and TC48. At TC35, there was a wide frequency distribution of chromosome numbers extending from triploid to hexaploid, the modal number being 75 (22%). However, by TC48, chromosome numbers were largely restricted to the triploid area, with 4% of cells in the tetraploid and only one (2%) in the hexaploid range. The modal number had shifted from 75 to 72 (22%) with two other peaks at 71 (16%) and 75 (14%).

In 38% of the cells at TC48, an additional minute chromosome per cell was observed. About 90% of cells in all *in vitro* passages examined contained 2Y chromosomes, although metaphases prepared from the patient's phytohaemagglutinin-stimulated blood lymphocytes showed the normal diploid male pattern. Table II gives a detailed study of the distribution of the Y chromosome in J82 cells at TC48.

*Karyotype.*—Detailed karyotypic ana-

FIG. 5.—A loose arrangement of cytoplasmic (tono) filaments in a cultured J82 cell. An area of annulate lamellae is located at the arrow. $\times$ 20,000.

lysis by quinacrine fluorescence banding was performed at TC48 (Fig. 8 and 9). Chromosome Groups A and B had numerical changes. Chromosomes 1 and 4 appeared normal, and Chromosomes 2, 3 and 5 were generally trisomic. C and D chromosomes had extensive numerical changes. Chromosomes 6, 7, 9, 11 and 15 were usually trisomic. In some metaphases, Chromosomes 7 and 11 had *two* extra homologues. Characteristically, Chromosome 13 had 5 homologues.

Groups E, F and G also had abnormal chromosome numbers. Chromosomes 17 and 18 were trisomic, 19 and 20 sometimes had 4 homologues, and 22 often showed trisomy. Chromosomes 8, 10, 14 and 21 generally appeared normal. Chromosome 21 had a bright satellite. Most of the cells had two X chromosomes.

*Marker chromosomes.*—At least 5, and frequently 7 or more, abnormal chromosomes have been identified in these cells on the basis of their characteristic banding pattern. These markers were not the same as the HeLa cell markers described by Nelson-Rees *et al.* (1974).

*HLA.*—The patients leucocytes typed for HLA A2, AW32, B5, B12. The cell line J82 at *in vitro* Passage 12 typed for HLA AW32 and B5 only.

*Lymphocyte cytotoxicity*

Cytotoxicity by autologous peripheral-blood lymphoid cells from the donor of J82 cells was tested on 8 occasions during a 3-year period. Table III shows the results of sequential tests using a microplate assay and visual counting. At each test the number of residual target cells remaining after incubation with the patient's lymphoid cells was compared to that after incuba-

TABLE I.—*Chromosome frequency distribution in J82 cells*

| *In vitro* passage | Chromosome number | | | | | | | | | | | | | | | | | | | | | | | | | | |
|---|---|---|---|---|---|---|---|---|---|---|---|---|---|---|---|---|---|---|---|---|---|---|---|---|---|---|
| | 65 | 66 | 67 | 68 | 69 | 70 | 71 | 72 | 73 | 74 | 75 | 76 | 77 | 78 | 79 | 80 | 82 | 83 | 87 | 91 | 96 | 100 | 110 | 118 | 130 | 144 | n |
| 35 | | | | | | 1 | 2 | 2 | 1 | 2 | 9 | 2 | 5 | 3 | 2 | 2 | 3 | | 1 | 1 | | 1 | 1 | 1 | 1 | | 41 |
| 48* | 2 | 1 | 1 | 3 | 3 | 6 | 8 | 11† | 1 | 2 | 7 | 1 | 1 | | | | | 1 | | | 1 | | | | | 1‡ | 50 |

FIG. 6.—The cytoplasm of some J82 cells contained numerous tightly packed bundles of filaments. ×1200.

tion with control donors' lymphocytes, and also with the number of surviving target cells incubated without effector cells (medium control). The same pattern of reactivity by J82 lymphoid cells was found using either control comparison. Before therapy the patient's lymphoid cells showed a preferential reaction against the allogeneic TCC cell line T24 as compared to HCV-29 cells. During the course of radiotherapy, after 1950 rad the patient reacted no differently from a control donor against two allogeneic cell lines derived from TCC and melanoma. Two weeks after 4200 rad the patient's lymphoid cells reacted significantly against HCV-29 cells. Three weeks after cystectomy, the patient showed no significant reaction against allogeneic cells. By 4 and 6 months after treatment, however, cytotoxicity was mainly restricted to autologous cells (J82) and the allogenic TCC target T24. One and a half years after surgery, no significant differences in cytotoxic activity between the patient and a control donor was observed on either the autologous, or 4 different allogenic cell lines. Three years after surgery, the patient's lymphocytes were tested for cytotoxicity in a $^{51}Cr$-release assay (Table IV). At this time, no significant cytotoxicity was observed against autologous or allogeneic cells as compared with a healthy person's lymphocytes or those from 2 patients cured of melanoma.

### DISCUSSION

Cytogenetic analysis of fresh bladder-carcinoma specimens have usually indicated a bimodal chromosome pattern (Atkin,

FIG. 7.—Chromosome frequency distribution in J82 cells at *in vitro* Passages 35 and 48.

TABLE II.—*Observation of metaphases with two Y chromosomes at* in vitro *Passage 48*

| No. of Ys | No. of cells |
|---|---|
| 2 | 36 (90·0%) |
| 1 | 3 (7·5%) |
| 0 | 1 (2·5%) |
| Total | 40 |

1976). Spooner and Cooper (1972), in a study of 61 bladder carcinomas, found that differentiated tumours had peridiploid karyotypes. In contrast, 80% of poorly differentiated tumours showed chromosome numbers in the triploid–hypertetraploid range. Similarly Sandberg (1977), in a comparative study of surgical specimens from papillary or invasive bladder carcinomas, drew a comparison between the degree of malignancy and the presence of extra abnormal marker chromosomes. Noninvasive papillary lesions karyotyped in the diploid area, while advanced tumours were triploid or tetraploid. The cell line J82 presented here had a broad chromosome distribution with abnormal banding patterns after short-term and long-term *in vitro* culture. Although this tumour was not karyotyped before culture, these results are consistent with invasive TCC.

In view of the persistent problem of cross-contamination of cell cultures by other rapidly growing lines (Nelson-Rees *et al.*, 1974) it is necessary to establish the identity of each new cell line. The presence of two Y chromosomes in the majority of J82 cells provides convincing evidence that these cells are of male origin. Duplicated Y bodies have been reported earlier in bladder carcinomas with high chromosome numbers (Atkin, 1973). Two Y chromosomes were also noted in an invasive TCC by Sandberg (1977). We have previously described a cell line from a squamous carcinoma of human bladder in which 2 Y bodies were found (O'Toole *et al.*, 1976). Metaphases prepared from blood lymphocytes of the donor of J82 cells showed a normal diploid male karyotype. Thus the abnormal chromosome patterns found in the J82 cell line probably reflect neoplasia-related changes, and are consistent with undifferentiated TCC. The fact that two Y bodies were found from *in vitro* Passage 5 onwards shows that this is a stable feature of this cell line.

The morphology of the J82 line is consistent with undifferentiated TCC; the cells show multilayering and lack of contact inhibition. Similar observations have been reported on these cells by Marshall *et al.* (1977). The enzymatic phenotype of the J82 line has been reported by Povey *et al.* (1976) to differ from HeLa and the other long-term cultures of TCC, T24 (Bubenik *et al.*, 1973) and RT4 (Rigby and Franks, 1970).

The long-term tumour-free status of the donor of J82 cells after treatment has permitted sequential studies on peripheral-blood lymphoid-cell cytotoxicity against both autologous and allogeneic tumour cells. Cytotoxicity towards autologous tumour was observed in early-passaged cells. Simultaneously, cytotoxicity was detected on allogeneic TCC targets from the

FIG. 8.—Q-banding karyotype of J82 cells at 48th passage, showing 74 chromosomes. Chromosomes are XXYY, +2, +3, +5, +6, ++7, +9, +11, +12, +++13, +15, +17, +18, +19, ++20, +22 and 7 markers (M1–M6).

TABLE III.—*Cytotoxicity of lymphoid cells to autologous and allogeneic target cells tested in microplate assays*

| Clinical situation at test | Target cells | E:T[1] | MC[2] | Surviving targets/well (mean ± s.d.) Patient (J82) | Control[3] (C) | % Reduction J82[4] | C[4] | J82[5] |
|---|---|---|---|---|---|---|---|---|
| | | | | | C161 | | | |
| Untreated | T24 | 500:1 | 103±16 | 33±7 | 92±17 | 68 | 11 | 64*** |
| | | 250:1 | | 55±16 | 87±15 | 47 | 16 | 37*** |
| | HCV-29 (TC33) | 500:1 | 39±9 | 28±5 | 23±6 | 28 | 41 | 0 |
| | | 250:1 | | 31±8 | 26±8 | 21 | 33 | 0 |

TABLE III.—*contd.*

| Clinical situation at test | Target cells | E:T[1] | MC[2] | Surviving targets/well (mean ± s.d.) Patient (J82) | Control[3] (C) | % Reduction J82[4] | C[4] | J82[5] |
|---|---|---|---|---|---|---|---|---|
| 1950 rad | | | | | J95 | | | |
| | T24 | 500:1 | 56±12 | 42±8 | 43±10 | 25 | 23 | 2 |
| | | 250:1 | | 51±10 | 55±16 | 9 | 2 | 7 |
| | MEL-1 (TC1) | 500:1 | 30±5 | 28±8 | 32±4 | 7 | 0 | 12 |
| | | 250:1 | | 26±6 | 28±6 | 13 | 7 | 7 |
| 4200 rad +2-week pause | | | | | J138 | | | |
| | T24 | 500:1 | 34±11 | 24±9 | 27±6 | 29 | 21 | 11 |
| | | 250:1 | | 30±10 | 35±6 | 12 | 0 | 14 |
| | HCV-29 (TC36) | 500:1 | 36±10 | 26±4 | 38±10 | 28 | 0 | 32* |
| | | 250:1 | | 30±8 | 50±16 | 17 | 0 | 40* |
| Post cystectomy | | | | | C184 | | | |
| | T24 | 500:1 | 102±17 | 83±16 | 90±16 | 19 | 12 | 8 |
| | | 250:1 | | 94±19 | 115±20 | 8 | 0 | 18 |
| 3 weeks | HCV-29 (TC44) | 500:1 | 36±10 | 34±10 | 32±10 | 6 | 11 | 0 |
| | | 250:1 | | 34±12 | 32±8 | 6 | 11 | 0 |
| 4 months | | | | | J195 | | | |
| | T24 | 500:1 | 183±32 | 79±19 | 202±24 | 57 | 0 | 61*** |
| | | 250:1 | | 152±37 | 203±16 | 17 | 0 | 25*** |
| | J82+ (TC1) | 500:1 | 189±24 | 130±29 | 185±12 | 31 | 2 | 30*** |
| | | 250:1 | | 169±23 | 193±8 | 11 | 0 | 12** |
| | J82+ (TC3) | 500:1 | 54±7 | 32±11 | 46±12 | 41 | 15 | 30** |
| | | 250:1 | | 30±5 | 53±8 | 44 | 2 | 43*** |
| | MEL-1 (TC13) | 500:1 | 194±12 | 187±18 | 201±17 | 4 | 0 | 7 |
| | | 250:1 | | 188±17 | 196±7 | 3 | 0 | 4 |
| 6 months | | | | | C216 | | | |
| | J82+ (TC10) | 500:1 | 59±9 | 31±6 | 49±10 | 47 | 17 | 37*** |
| | | 250:1 | | 39±9 | 52±6 | 34 | 12 | 25*** |
| | HCV-29 (TC58) | 500:1 | 36±10 | 26±9 | 28±8 | 28 | 22 | 7 |
| | | 250:1 | | 36±15 | 36±10 | 0 | 0 | 0 |
| 18 months | | | | | C253 | | | |
| | T24 | 500:1 | 90±14 | 88±13 | 71±15 | 0 | 21 | 0 |
| | | 250:1 | | 109±15 | 103±16 | 0 | 0 | 0 |
| | J82+ (TC33) | 500:1 | 61±18 | 66±13 | 41±11 | 0 | 33 | 0 |
| | | 250:1 | | 77±11 | 59±12 | 0 | 3 | 0 |
| | RT4 | 500:1 | 103±21 | 68±20 | 61±19 | 34 | 41 | 0 |
| | | 250:1 | | 95±9 | 99±13 | 8 | 4 | 4 |
| | HCV-29 (TC80) | 500:1 | 115±30 | 100±20 | 95±16 | 13 | 17 | 0 |
| | | 250:1 | | 102±15 | 106±21 | 11 | 8 | 4 |
| | MEL-1 (TC25) | 500:1 | 25±9 | 20±8 | 21±6 | 20 | 16 | 5 |
| | | 250:1 | | 28±7 | 26±6 | 0 | 0 | 0 |

1 Effector: target cell ratio at beginning of incubation.
2 Medium control, targets incubated with medium alone.
3 J95 Cutaneous melanoma. J138 Cystitis. J195 Adenocarcinoma bladder. C161, C184, C216, C253 Normal healthy controls.
4 Relative to medium control (MC)
5 Relative to control effector cells (C).
+ Autologous tumour cells
* $P<0.05$. ** $P<0.01$. *** $P<0.001$.

TABLE IV.—*Lymphoid-cell cytotoxicity against autologous and allogeneic targets measured by $^{44}Cr$ release*

| Effector cells | E:T | % isotope release from targets* T24 | J82† TC50 | HCV-29 TC107 | MEL-1 TC46 |
|---|---|---|---|---|---|
| J308 Melanoma | 100:1 | 5 | 3 | 6 | 2 |
| | 50:1 | 4 | 2 | 3 | 1 |
| J309 Melanoma | 100:1 | 3 | 2 | 7 | 2 |
| | 50:1 | 2 | 0 | 3 | 2 |
| J82 3 years | 100:1 | 9 | 1 | 8 | 7 |
| post-cystectomy | 50:1 | 4 | 0 | 3 | 5 |
| Normal healthy | 100:1 | 3 | 1 | 1 | 1 |
| | 50:1 | 1 | 1 | 1 | 0 |

* Corrected for spontaneous isotope release: T24=16; J82=21; HCV-29=17; MEL-1=27. Incubation time 24 h.
† Autologous tumour cells.

FIG. 9.—Q-banding karyotype of J82 cell at 48th passage with 65 chromosomes. Chromosomes are XY, +2, +3, +5, +6, +7, +9, ++11, +++13, +17, +18, and 6 markers (Ml–M5).

line T24. Significant cytotoxicity to both autologous and allogeneic (T24) TCC targets was found 4 and 6 months after the patient had received preoperative radiation and cystectomy, but was lost by 18 months and 3 years. These results concur with previous sequential studies on cytotoxicity toward allogeneic TCC targets in patients given preoperative radiation and cystectomy (O'Toole *et al.*, 1973). It should be noted that, 2 weeks after radiotherapy, cytotoxicity was observed on the allogeneic cell line HCV-29 derived from urothelium. However, this reaction was transient.

J82 cells have been tested routinely during the last 5 years as targets for allogeneic lymphoid-cell cytotoxicity. Effector cells from some patients with localized TCC were cytotoxic toward primary cultured J82 cells (O'Toole *et al.*, 1974; Nayak *et al.*, 1977). However, after long-term *in vitro* culture this type of reaction was no longer detected (O'Toole, 1977). Throughout this observation period, allogeneic cytotoxicity by TCC patients' lymphoid cells was detected on the cell line T24 derived from TCC. These data suggest that the J82 cells have undergone antigenic changes during prolonged *in vitro* culture.

This study was supported by Grants CA 20216 awarded by the National Cancer Institute, DHEW, and 73213 from the Swedish Cancer Society. Y. Ohnuki wishes to acknowledge Miss M. Marnell for expert technical assistance and E. C. Read and R. S. Olson for photographic processing.

### REFERENCES

ATKIN, N. B. (1973) Y bodies and similar fluorescent chromocenters in human tumours including teratoma. *Br. J. Cancer*, **27**, 183.

ATKIN, N. B. (1976) *Cytogenetic Aspects of Malignant Transformation.* Basel, New York: S. Karger. p. 92.

BUBENIK, J., BARESOVA, M., VIKLICKY, C., JAKOUBKOVA, J., SAINEROVA, H. & DONNER, J. (1973) Established cell line of urinary bladder carcinoma (T24) containing tumor-specific antigen. *Int. J. Cancer*, **11**, 765.

KUMEGAWA, M., CATTONI, M. & ROSE, G. G. (1967) Electron microscopy of oral cells *in vitro*. I. Annulate lamellae observed in strain KB cells. *J. Cell Biol.*, **34**, 897.

KUMEGAWA, M., CATTONI, M. & ROSE, G. G. (1968) Electron microscopy of oral cells *in vitro*. II. Subsurface and intracytoplasmic confronting cisternae in strain KB cells. *J. Cell Biol.*, **36**, 443.

MARSHALL, C. J., FRANKS, L. M. & CARBONELL, A. W. (1977) Markers of neoplastic transformation in epithelial cell lines. *J. Natl. Cancer Inst.*, **58**, 1743.

NAYAK, S. K., O'TOOLE, C. & PRICE, Z. H. (1977) A cell line from an anaplastic transitional cell carcinoma of human urinary bladder. *Br. J. Cancer*, **35**, 142.

NELSON-REES, W. A., FLANDERMEYER, R. R. & HAWTHORNE, R. K. (1974) Banded marker chromosomes as indicators of intraspecies cellular contamination. *Science*, **184**, 1093.

O'TOOLE, C. (1977) A $^{51}$chromium isotope release assay for detecting cytotoxicity to human bladder carcinoma. *Int. J. Cancer*, **19**, 324.

O'TOOLE, C., NAYAK, S., PRICE, Z., GILBERT, W. H. & WAISMAN, J. (1976) A cell line (SCaBER) derived from squamous cell carcinoma of the human urinary bladder. *Int. J. Cancer*, **17**, 707.

O'TOOLE, C., STEJSKAL, V., PERLMANN, P. & KARLSSON, M. (1974) Lymphoid cells mediating tumor-specific cytotoxicity to carcinoma of the urinary bladder: separation of the effector population using a surface marker. *J. Exp. Med.*, **139**, 457.

O'TOOLE, C., UNSGAARD, B., ALMGÅRD, L. E. & JOHANSSON, B. (1973) The cellular immune response to carcinoma of the urinary bladder: correlation to clinical stage and treatment. *Br. J. Cancer*, **28** (Suppl. 1), 266.

POVEY, S., HOPKINSON, D. A., HARRIS, H. & FRANKS, L. M. (1976) Characterization of human cell lines and differentiation from HeLa by enzyme typing. *Nature*, **264**, 60.

RIGBY, C. C. & FRANKS, L. M. (1970) A human tissue culture cell line from a transitional cell tumor of the urinary bladder: Growth, chromosome pattern and ultrastructure. *Br. J. Cancer*, **24**, 746.

RUSSELL, W. C., NEWMAN, C. & WILLIAMSON, D. H. (1975) A simple cytochemical technique for demonstration of DNA in cells infected with mycoplasmas and virus. *Nature*, **253**, 461.

SANDBERG, A. A. (1977) Chromosome markers and progression in bladder cancer. *Cancer Res.*, **37**, 2950.

SEMAN, G. & DMOCHOWSKI, L. (1975) Ultrastructural characteristics of human tumor cells *in vitro*. In *Human Tumor Cells in vitro*. Ed. J. Fogh. New York: Plenum Press. p. 395.

SOFEN, H. & O'TOOLE, C. (1978) Anti-squamous tumor antibodies in patients with squamous cell carcinoma. *Cancer Res.*, **38**, 199.

SPOONER, M. E. & COOPER, E. H. (1972) Chromosome constitution of transitional cell carcinoma of the urinary bladder. *Cancer*, **29**, 1401.

UCHIDA, I. A. & LIN, C. C. (1974) Quinacrine fluorescent patterns. In *Human Chromosome Methodology*. Ed. J. J. Yunis. New York: Academic Press. p. 47

UNSGAARD, B. & O'TOOLE, C. (1975) The influence of tumour burden and therapy on cellular cytotoxicity responses in patients with ocular and skin melanoma. *Br. J. Cancer*, **31**, 301.